# Airway Pressure Release Ventilation with a Time-Controlled Adaptive Ventilation Strategy (APRV-TCAV) Reverses Endotracheal Secretion Movement Compared with Volume-Control Ventilation: A Bench Study

**DOI:** 10.3390/jcm15166176

**Published:** 2026-08-10

**Authors:** Ben T. Daxon, William M. LeTourneau, Brendan T. Wanta, Mariah L. Bennett, Abier M. Dawood, Rylee N. Stewart, Pia P. McEleney

**Affiliations:** 1 Department of Anesthesiology and Perioperative Medicine, Mayo Clinic, 200 First Street SW, Rochester, MN 55905, USA; 2 Department of Respiratory Care, Mayo Clinic, 200 First Street SW, Rochester, MN 55905, USA; letourneau.william@mayo.edu (W.M.L.);

**Keywords:** airway pressure release ventilation, APRV, time-controlled adaptive ventilation, TCAV, personalized mechanical ventilation, endotracheal tube, airway secretion transport, surrogate secretion movement, airway secretions, bench model

## Abstract

**Background/Objectives:** Retained airway secretions are a common complication of mechanical ventilation and a major contributor to ventilator-associated pneumonia. Airway Pressure Release Ventilation following a Time-Controlled Adaptive Ventilation protocol (APRV-TCAV) has been anecdotally observed to improve secretion clearance, but the mechanism is not well established. Because TCAV sets the release time (TLow) from each patient’s own expiratory flow decay, we hypothesized that the resulting expiratory-dominant flow profile would generate a net antegrade force on secretions. This bench study compared secretion movement between APRV-TCAV and conventional Volume-Control Continuous Mandatory Ventilation (VC-CMV), with and without an in-line oscillatory device. **Methods:** A critical care ventilator was connected to a 3 L test lung via a 7 mm endotracheal tube (ETT). Simulated mucus (1% guar gum) was instilled into the ETT, and secretion movement was measured under VC-CMV (positive end-expiratory pressure (PEEP) 5, 10, 15 cmH_2_O) and APRV-TCAV (PHigh 20, 25, 30 cmH_2_O; PLow 0 cmH_2_O). Each condition was repeated with an in-line oscillatory device. **Results:** All VC-CMV settings produced retrograde secretion movement toward the test lung; all APRV-TCAV settings produced antegrade movement. The largest antegrade movement was observed at the widest PHigh-to-PLow differential. Adding the oscillatory device raised peak expiratory flow above peak inspiratory flow in every condition, yet abolished antegrade movement under APRV-TCAV. **Conclusions:** In this bench study, APRV-TCAV reversed the direction of secretion movement within the ETT, with larger antegrade movement at wider PHigh-to-PLow differentials. This suggests a potential mechanism by which the ventilator flow profile itself may influence secretion movement. The directional effect depended on the intact release-phase waveform and was abolished by in-line oscillatory therapy despite preserved peak flow asymmetry.

## 1. Introduction

Airway Pressure Release Ventilation (APRV) is a mode of mechanical ventilatory support characterized by a prolonged high-pressure phase (PHigh) for lung recruitment, interrupted by a very brief, time-cycled release phase to a low pressure (PLow) for ventilation. Within APRV, the Time-Controlled Adaptive Ventilation (TCAV) protocol personalizes and adapts this mode patient-by-patient by setting the release time (TLow) based on the lung’s own expiratory flow characteristics, terminating the release when expiratory flow has decayed to 75% of its peak [1]. Because TLow is derived from the patient’s instantaneous lung mechanics rather than set arbitrarily, the delivered tidal volume and residual end-expiratory pressure adjust automatically as the patient improves or worsens. This results in a mechanically unique “upside-down” ventilation profile where expiratory flows exceed inspiratory flows [2].

This concept becomes important in mechanically ventilated patients, where the accumulation of respiratory secretions is a common complication that contributes to airway obstruction, atelectasis, and ventilator-associated pneumonia (VAP) [3]. The endotracheal tube (ETT) is central to this problem: it disrupts the mucociliary escalator and provides a surface on which secretions collect, yet how the ventilator mode itself influences the movement of those secretions within the ETT has received little attention.

A possible link between APRV and reduced VAP risk has been suggested in prior clinical work: in a retrospective cohort of trauma patients with pulmonary contusion, Walkey et al. found a significantly lower incidence of VAP with APRV than with conventional ventilation, which they attributed to improved lung recruitment and reduced sedation [4]. Our study examines an additional, complementary possibility: a direct influence of the APRV breath profile on the directional movement of airway secretions within the ETT.

In conventional mechanical ventilation such as Volume-Controlled Continuous Mandatory Ventilation (VC-CMV), inspiration is a fixed process with ventilator-driven flow, while expiration is passive. This often results in higher peak inspiratory flows than expiratory flows, creating a net force that may drive secretions deeper into the airways. In contrast, APRV, when set with the TCAV protocol, generates a unique flow differential [5]. The large pressure gradient created by cycling from a high PHigh to a low PLow of zero produces a high-velocity expiratory flow. This results in faster expiratory flows than inspiratory flows, creating a net outward force that clinicians have anecdotally observed to be more effective at expelling secretions [6]. Although this strong mechanical rationale has been proposed, direct experimental evidence confirming this effect has been limited.

We theorized that APRV-TCAV promotes antegrade movement of the surrogate fluid during the release phase from a high PHigh to a PLow of zero: the large pressure gradient generates a high-velocity expiratory flow, while the return to PHigh occurs against residual intrinsic lung pressure, producing a smaller inspiratory gradient and lower inspiratory flow. This flow differential, we propose, creates a net outward shear force. The objective of this study was to compare the direction and magnitude of surrogate secretion movement within an ETT between APRV-TCAV and VC-CMV, and to examine the effect of an in-line oscillatory device.

## 2. Materials and Methods

**Figure 1:**
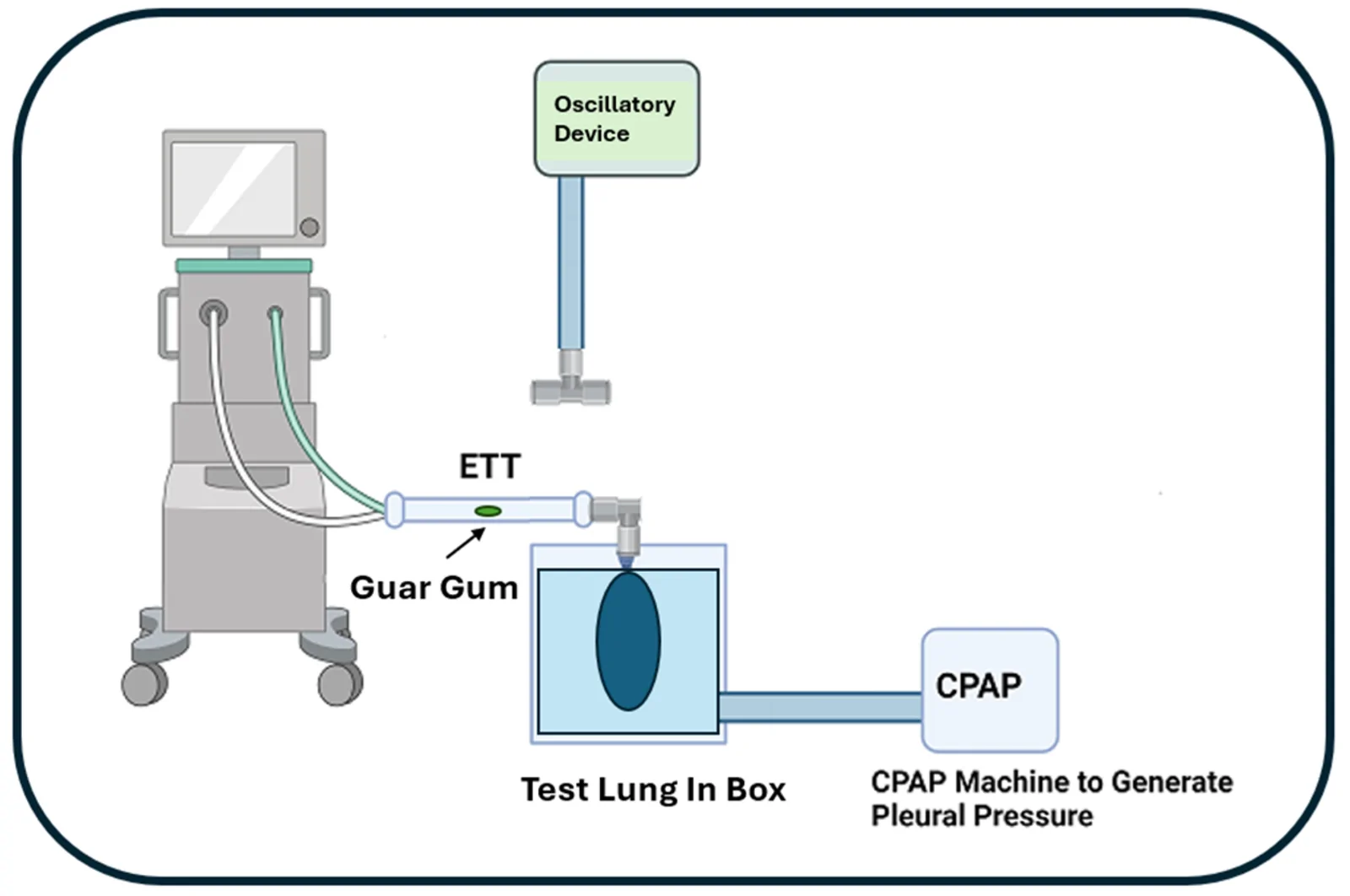
Bench model for assessment of secretion movement within the endotracheal tube.

**Figure 2:**
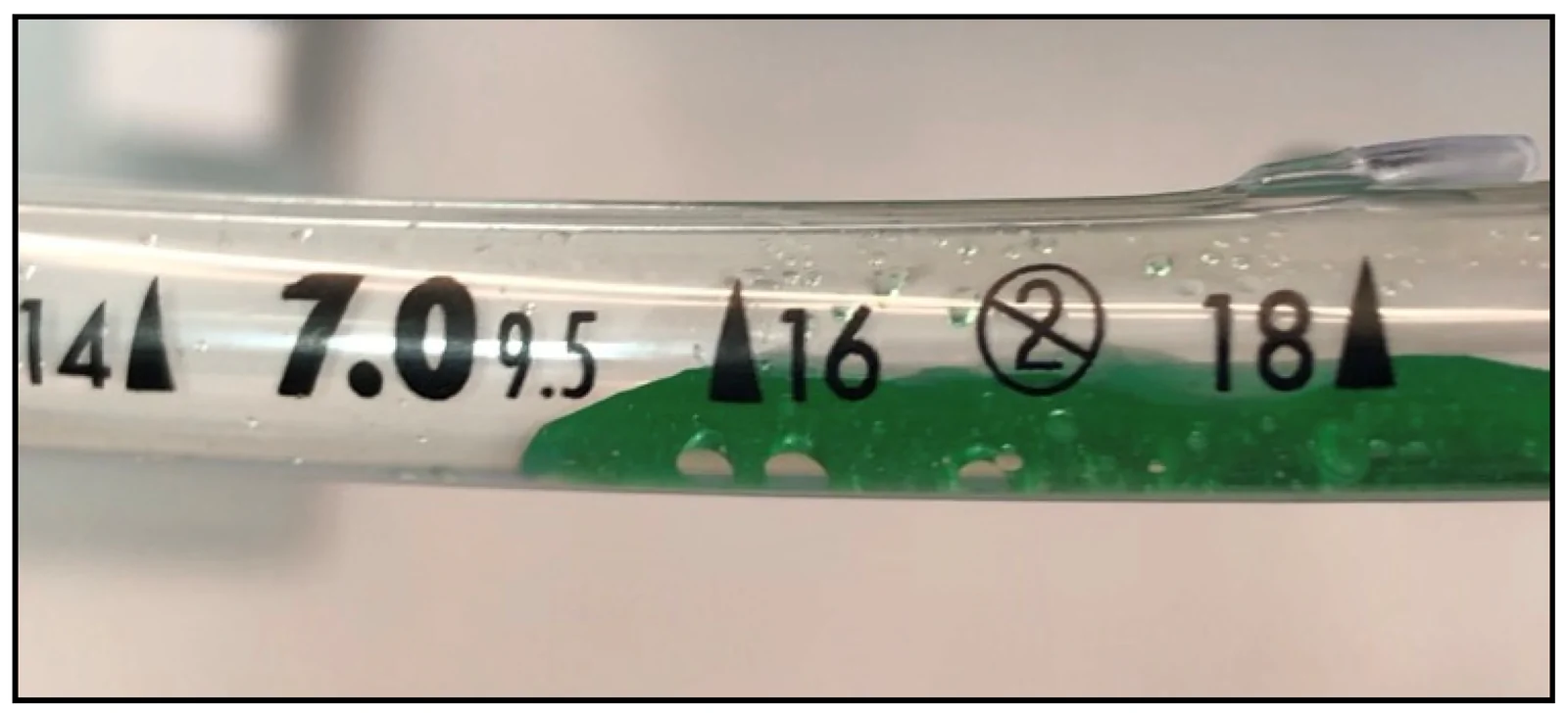
Guar gum simulating mucus inside an endotracheal tube.

A simplified single-compartment lung model was constructed to reproduce the pressure–flow relationships of mechanical ventilation (Figure 1). A critical care ventilator (Hamilton Medical (G5); Bonaduz, Switzerland) was connected to a 3 L test lung (AirLife; Grand Rapids, MI, USA) housed within an acrylic box. Pleural pressure was simulated by connecting the acrylic box to a continuous positive airway pressure (CPAP) circuit (ResMed (S9); San Diego, CA, USA), which maintained a constant intra-box pressure throughout each ventilator cycle. Box pressure was set independently for each ventilator condition to maintain expiratory transpulmonary pressure between −2 and +2 cmH_2_O. A 7 mm endotracheal tube (ETT) (Covidien; Shiley 7.0 mm ETT; Mansfield, MA, USA) was placed horizontally between the ventilator circuit and the test lung. Simulated sputum was prepared using a 1% guar gum solution (1 g guar gum per 100 mL sterile water), consistent with the recipe used by Mahajan et al. (Figure 2) [6]. An in-line oscillatory device was available for testing its additive effects.

### Ventilator Settings

- VC-CMV: Fixed settings included a respiratory rate of 16 breaths/minute, a tidal volume of 300 mL, and an inspiratory flow of 40 L/min. Positive end-expiratory pressure (PEEP) was tested at 5, 10, and 15 cmH_2_O.
- APRV-TCAV: Fixed settings included a THigh of 4.0 s and a PLow of 0 cmH_2_O. The TLow was set to terminate expiration at 75% of peak expiratory flow (0.2–0.3 s). PHigh was tested at 20, 25, and 30 cmH_2_O.

In each experiment, 3 mL of simulated mucus was instilled into the ETT’s midpoint. Movement of the proximal edge of the mucus was measured in millimeters per minute (mm/min). Movement away from the lung was denoted as positive, and movement toward the lung as negative. Each condition was observed for 2 min and repeated 3 times. This duration produced measurable displacement while remaining short enough that mucus rheology and humidification status would not be appreciably altered.

Simulated pleural pressures were adjusted according to set PEEP in VC-CMV and measured PEEP in APRV-TCAV to maintain an expiratory transpulmonary pressure between −2 and +2 cmH_2_O.

A MetaNeb oscillatory device (Hillrom Inc., Chicago, IL, USA) was inserted in-line in the inspiratory limb of the ventilator circuit via spring-valve tee, proximal to the endotracheal tube (ETT). A second set of measurements was performed for all previously tested ventilator settings for both VC-CMV (PEEP 5, 10, and 15 cmH_2_O) and APRV-TCAV (PHigh 20, 25, and 30 cmH_2_O). Oscillations were delivered continuously throughout the entire respiratory cycle—during both THigh and TLow—for the full duration of each device-on measurement. The device was operated using the manufacturer’s standard clinical settings used at our institution (Mode: CHFO, “Higher” percussion rate and pressure, and resister ring bypassed); oscillation frequency and amplitude were not independently measured.

Secretion movement was measured in the same manner as the initial experiments. Because only three replicates were performed per condition, results are reported as mean ± standard deviation and summarized descriptively; formal statistical testing was not performed.

## 3. Results

### 3.1. Secretion Movement Without an Oscillatory Device

**Figure 3:**
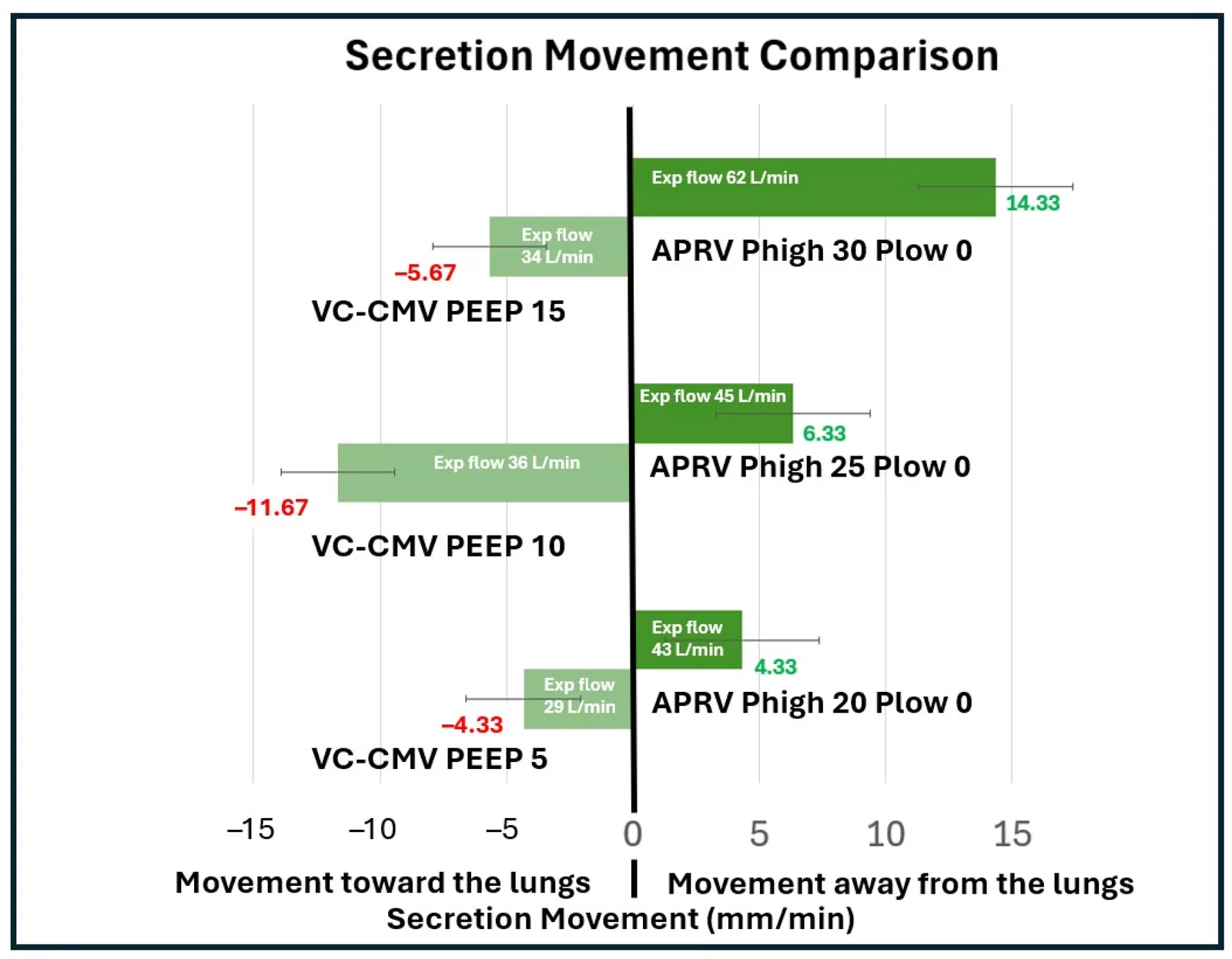
Comparison of secretion movement. APRV vs. VC-CMV. No oscillatory device in line.

The direction of secretion movement was determined by the ventilation mode (Figure 3). In all three VC-CMV settings, secretions moved retrograde (toward the test lung). In all three APRV-TCAV settings, secretions moved antegrade (away from the test lung). The two modes separated completely, with no overlap: every VC-CMV condition produced retrograde movement and every APRV-TCAV condition produced antegrade movement, so the central finding did not depend on statistical testing.

- Under VC-CMV with a fixed inspiratory flow of 40 L/min, peak expiratory flow was lower than peak inspiratory flow at every PEEP tested. Mucus moved retrograde at PEEP 5 (−4.33 ± 2.87 mm/min; expiratory flow 29 L/min), PEEP 10 (−11.67 ± 7.41 mm/min; 36 L/min), and PEEP 15 (−5.67 ± 0.94 mm/min; 34 L/min).
- Under APRV-TCAV with PLow 0 and TLow set to terminate at 75% of peak expiratory flow, peak expiratory flow exceeded peak inspiratory flow at every PHigh tested. Mucus moved antegrade at PHigh 20 (+4.33 ± 4.19 mm/min; inspiratory/expiratory flow 40/43 L/min), PHigh 25 (+6.33 ± 1.88 mm/min; 42/45 L/min), and PHigh 30 (+14.33 ± 6.64 mm/min; 52/62 L/min). Antegrade movement increased across the three PHigh settings as the expiratory-to-inspiratory flow difference widened. The largest antegrade movement was observed at PHigh 30, where the expiratory-to-inspiratory flow difference was also widest (62 vs. 52 L/min).

### 3.2. Effect of an In-Line Oscillatory Device

**Figure 4:**
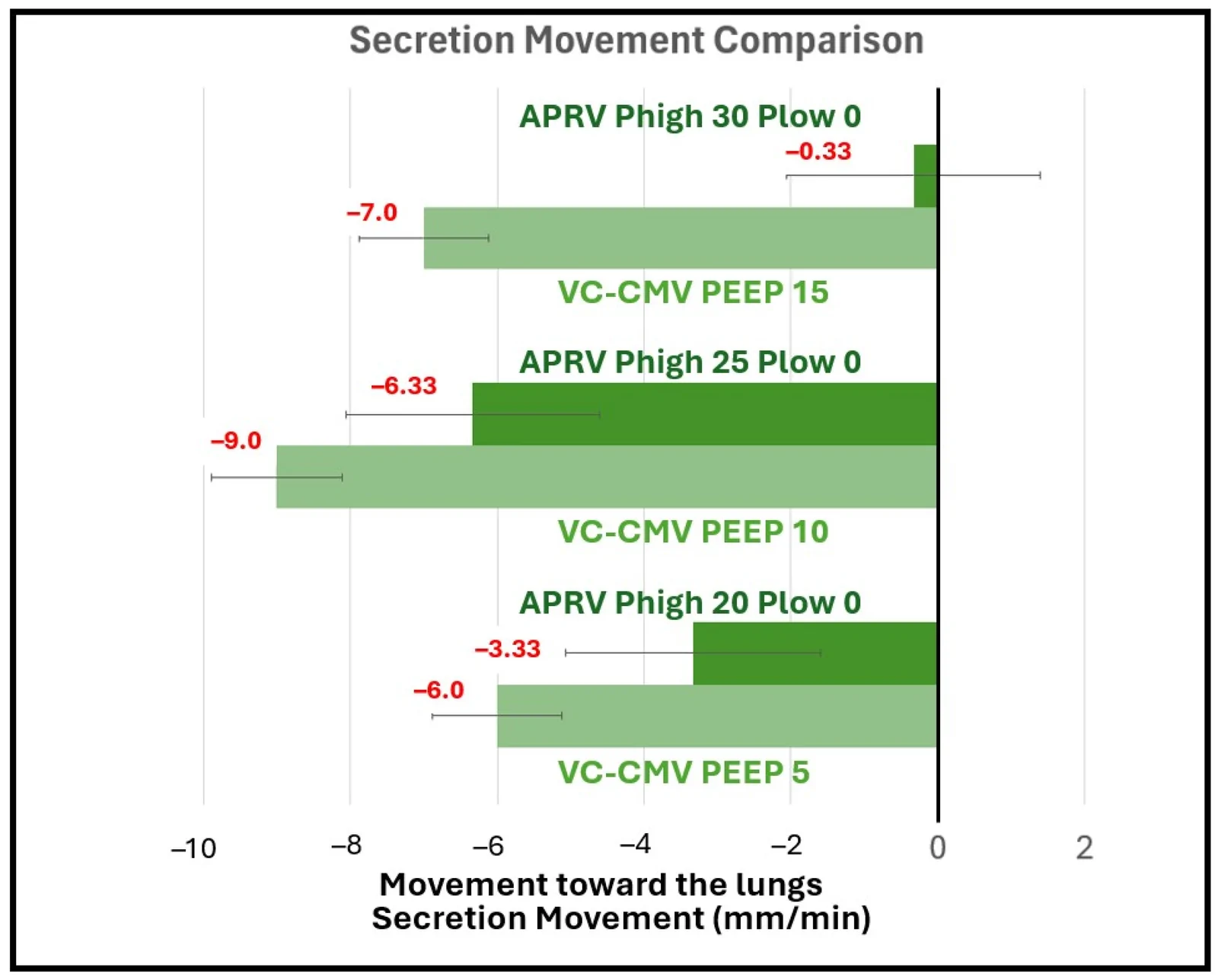
Comparison of secretion movement. APRV vs. VC-CMV. Oscillatory device in line.

The application of an in-line oscillatory device did not alter the direction of secretion movement in VC-CMV, though it did slightly reduce the magnitude of the retrograde movement (Figure 4). The device did, however, completely prevent antegrade movement with APRV-TCAV. In two of the three settings (APRV PHigh 20 and 25), this resulted in a slight retrograde movement, while the setting with the highest PHigh-to-PLow differential (PHigh 30) showed a negligible net movement. Notably, the device did not abolish the expiratory-dominant flow profile: under APRV, the peak expiratory–to–inspiratory flow differential was preserved or amplified with the device in line, and under VC-CMV the device raised peak expiratory flow above peak inspiratory flow at every PEEP (Table 1). Despite this, antegrade movement was abolished in every condition. The oscillatory device also altered measured end-release airway pressure under APRV-TCAV (8.5, 12.0, and 11.3 cmH_2_O at PHigh 20, 25, and 30 with the device, vs. 10.0, 13.5, and 17.9 cmH_2_O without), consistent with disruption of the expiratory flow waveform that drives the antegrade effect.

## 4. Discussion

In this bench model, the expiratory-dominant flow profile of APRV-TCAV reversed the direction of surrogate fluid movement within the ETT, producing antegrade movement toward the proximal tube. This contrasts with our findings for conventional ventilation, where the surrogate fluid consistently moved retrograde toward the test lung. This observation is consistent with previous work by Mahajan et al., who reported that simulated mucus movement was closely related to peak expiratory flow when expiratory flow exceeded inspiratory flow [6].

Antegrade movement increased with the PHigh-to-PLow differential in this model. Because expiratory recoil rises as compliance falls [7], this raises the hypothesis—untested here, as we did not vary compliance—that the directional effect could be greater in less compliant lungs.

A plausible mechanistic interpretation is that the directional effect observed here reflects the coupling between the release-phase pressure gradient and expiratory flow decay. In TCAV, TLow is set from the expiratory flow profile, and higher elastance would be expected to generate faster expiratory flows and more rapid flow decay. Although lung elastance was not varied in the present model, this framework raises the hypothesis that the secretion-movement effect may vary with patient-specific respiratory mechanics. Future studies using variable-compliance or biologic lung models will be needed to test whether this effect varies with lung injury severity or patient-specific respiratory mechanics.

The optimal setting for PLow in APRV remains a subject of ongoing debate [7,8,9,10,11,12]. We did not vary PLow. Prior bench work by Mahajan et al. [6] examined how PLow affects simulated secretion movement; rather than replicate that, we held PLow at zero and varied PHigh to isolate the effect of the pressure differential, and separately tested the in-line oscillatory device. Our data therefore cannot, on their own, identify an optimal PLow. A PLow of zero is nonetheless supported on mechanistic grounds and by prior literature: it maximizes the expiratory pressure gradient and, in our model, produced the highest expiratory flow velocities and the largest antegrade movement we observed.

This work also highlights the concept of PTRAP (Terminal Release Airway Pressure), the actual pressure remaining in the lung at the end of the TLow, which can be measured with an expiratory hold [13]. PTRAP was not a primary measurement in this study; we raise it as interpretive context from prior work [13,14]. This PTRAP, not the PLow setting, is the true end-expiratory pressure. The driving pressure in APRV-TCAV is therefore PHigh minus PTRAP. This concept is supported by the work of Daoud et al., who used esophageal manometry to measure transpulmonary pressures in APRV [14]. They demonstrated a profound discrepancy between the pressure displayed on the ventilator and the actual pressure within the lung; a negative end-expiratory transpulmonary pressure became positive after an expiratory hold was applied, revealing significant “trapped” pressure. This is analogous to the difference between peak inspiratory pressure and the lower, more physiologically relevant, plateau pressure in conventional ventilation, reinforcing the “upside-down” nature of APRV mechanics [2].

The findings regarding the in-line oscillatory device introduce a crucial consideration for clinical practice. While this device is designed to improve secretion clearance via high-frequency oscillations, our data suggest a complex interaction with the flow dynamics of APRV-TCAV. With the device in line, the retrograde movement observed in VC-CMV was slightly altered, but the antegrade movement seen with APRV-TCAV was completely prevented and, in some settings, reversed to retrograde flow.

This pattern was not explained by peak flow values. With the device in line, the expiratory-to-inspiratory flow differential was preserved or amplified under APRV, and under VC-CMV the device raised peak expiratory flow above peak inspiratory flow at every PEEP tested (Table 1). Yet despite peak expiratory flow exceeding peak inspiratory flow in every device-on condition, antegrade movement did not occur in any of them. The directional effect of APRV-TCAV on secretions therefore appears to depend not merely on peak flow asymmetry, but on the time-domain structure of the flow—the brief, sustained, unidirectional shear of the release phase. The high-frequency oscillations of the device fragment this shear into bidirectional flow excursions, abolishing the directional effect even when peak flow asymmetry is preserved.

Clinically, this suggests that combining an in-line oscillatory device with APRV-TCAV may negate rather than augment the secretion-movement benefit of either therapy alone. More broadly, if confirmed clinically, these findings would suggest that the choice of ventilator mode is itself a secretion-management decision, and that pairing APRV-TCAV with in-line oscillatory therapy may warrant reconsideration at the bedside. These findings should be regarded as hypothesis-generating: they are based on a single in-line oscillatory device tested across multiple ventilator settings, and whether other devices, alternative oscillatory frequencies, or different in-circuit positions would produce a similar attenuation is unknown. We caution against extrapolating this single-device observation to clinical decisions regarding oscillatory therapy, but suggest that the potential interaction between in-line oscillatory devices and APRV-TCAV merits further study.

This finding also sits within the broader context of VAP prevention, where the continuous, hands-off nature of APRV-TCAV offers a practical advantage. Subglottic secretion drainage (SSD), for example, removes secretions pooling above the ETT cuff—a major source of VAP [15]—but its effectiveness is limited by secretion viscosity and the need for frequent manual suctioning, which adds to nursing workload [16,17]. A recent overview of systematic reviews found that SSD significantly reduces VAP incidence (RR 0.56, 95% CI 0.48–0.63) and mortality (RR 0.88, 95% CI 0.80–0.97), though not duration of ventilation or length of stay [18]. By contrast, the directional effect of APRV-TCAV on secretions is integrated into the ventilator itself, providing a consistent, automated process that may reduce dependence on intermittent manual interventions.

This bench model does not measure VAP, mucociliary function, or clinical outcomes; the clinical literature summarized in Table 2 is offered only to frame hypotheses that a directional-flow mechanism might inform, not as evidence bearing on our results. The clearest VAP-specific signal comes from Walkey et al., a retrospective cohort of trauma patients with pulmonary contusion in which APRV use was associated with a substantial reduction in VAP risk after propensity adjustment (HR 0.10, 95% CI 0.02–0.58; *p* = 0.01) [4]. Notably, the APRV arm was sicker at baseline, with higher Lung Injury Scores and greater transfusion requirements—the latter an independent predictor of VAP—so the association is unlikely to reflect a lower-risk APRV population. The study is nonetheless small, retrospective, and not designed to isolate TCAV-specific settings or secretion-clearance mechanisms.

In the randomized trial by Maxwell et al., VAP episodes per patient were numerically higher in the APRV arm, but the difference was not significant and is difficult to attribute to ventilation mode: the APRV group had a worse APACHE II score (20.5 vs. 16.9, *p* = 0.027) and a trend toward lower GCS—an imbalance the authors acknowledged as a probable randomization error [20]. Sicker patients with more severe head injury would be expected to develop more VAP independent of mode.

The remaining studies are less directly informative, and in each the APRV strategy diverges from the TCAV profile examined here—most often through an elevated PLow that would attenuate the expiratory-dominant gradient central to our proposed mechanism. Gonzalez et al. pooled APRV and BIPAP as a single exposure (mean low pressure ~7 cmH_2_O); a lower VAP rate in the unmatched analysis (6% vs. 10%, *p* = 0.03) did not persist after propensity matching (6% vs. 9%, *p* = 0.15) and cannot be considered TCAV-specific [19]. The COVID-era reports share a common profile—APRV used largely as rescue therapy in sicker patients, with PLow elevated or unreported—and their VAP outcomes were null or not separately reported [21,22,23]. Separately, although VAP was not pre-specified, the Zhou ARDS trial (PLow 5 cmH_2_O) reported fewer ventilator days and reduced sedation with APRV, factors that could independently lower VAP risk, though these findings are debated [10].

This heterogeneous and sometimes conflicting evidence base is reflected in clinical guidelines, where APRV-TCAV is most often omitted or deferred rather than recommended, and at most positioned as an alternative to consider in refractory cases. Our findings add to a growing body of work suggesting that several mechanisms of APRV-TCAV remain underappreciated, and that a fuller mechanistic understanding may help explain the clinical signals already reported. Taken together, current clinical data do not establish that APRV or APRV-TCAV reduces VAP through enhanced secretion clearance; rather, they provide a hypothesis-generating context in which several non–mutually exclusive mechanisms—reduced sedation, preserved spontaneous breathing, shorter ventilator exposure, improved recruitment, and the directional flow-profile effect described here—could each contribute. A non-TCAV strategy that shortens ventilator exposure or lightens sedation may yield a benefit even where the flow-profile signature is attenuated; conversely, the directional reversal of secretion movement is most likely to occur when APRV is delivered as TCAV with a brief release phase and PLow at zero.

### Limitations

Our study, while demonstrating a clear directional effect on surrogate fluid movement in a simplified bench model, did not fully replicate the complex anatomy and physiology of the human respiratory system. Several factors limit the direct translation of these findings to a clinical setting. First, our intended preserved porcine lung model developed a leak prior to data collection, so we proceeded with a single-compartment 3 L test lung. While the test lung reliably reproduced the pressure–flow relationships necessary for our primary outcome of mucus movement within the ETT, it does not replicate the viscoelastic properties of biologic lung tissue, the airway compliance of native bronchi, or the dynamic interactions of the chest wall, and it limits the expiratory phase to a single time constant. This could affect the observed outcomes as it does not fully capture the physiological feedback loops present in a living patient.

We used a single 1% guar gum surrogate. Airway secretion rheology varies widely—cystic fibrosis secretions differ substantially from those in immunocompetent ARDS, and secretion composition evolves over the course of ARDS. Our surrogate was appreciably viscous but represents one rheologic state; more desiccated or more hydrated preparations may transit differently. Whether the directional effect holds across clinically encountered viscosities is unknown and warrants testing with a range of surrogate compositions. The oscillatory-device intervention was also tested using a single device and a single clinical operating configuration. Therefore, the findings should not be generalized to other oscillatory devices, alternative settings, or different circuit positions.

The inspiratory flow used in VC-CMV (40 L/min) is at the lower end of clinical practice; higher inspiratory flows would be expected to drive secretions further into the airway, magnifying rather than diminishing the directional contrast between modes. The per-breath nature of this effect was directly observable at the bench: simulated mucus migrated incrementally inward with each VC-CMV breath and incrementally outward with each APRV release. These considerations suggest our findings likely underestimate, rather than overstate, the magnitude of the directional effect. Future bench work should systematically vary inspiratory flow to better isolate the contribution of the expiratory-to-inspiratory flow ratio from total flow magnitude.

Finally, our model measured movement only within the ETT and could not track secretions in the distal airways and bronchi; we therefore demonstrated proximal movement but cannot confirm that it would translate to clearance from the entire respiratory tract. These limitations underscore the need for further investigation, including repeating these experiments with an intact biologic lung, dynamically varying simulated intrapleural pressure to better mimic spontaneous effort and chest wall mechanics, and instilling mucus within the trachea and proximal airways to determine whether the directional pattern seen in the ETT extends into the carina and main bronchi.

## 5. Conclusions

In this bench study, APRV-TCAV reversed the direction of secretion movement within the endotracheal tube, moving surrogate fluid away from the lung in every setting tested, while conventional ventilation moved it toward the lung. The size of this effect increased with the PHigh-to-PLow differential and depended on the intact release-phase waveform: an in-line oscillatory device abolished the outward movement even though peak expiratory-to-inspiratory flow asymmetry was preserved. These findings are specific to fluid movement within an endotracheal tube and should be considered hypothesis-generating. Whether the effect extends to secretions in the trachea and distal airways—and whether it ultimately reduces secretion burden or VAP in ventilated patients—will require confirmation in more anatomically faithful models and, eventually, clinical studies. If borne out, these results would suggest that the ventilator flow profile may be relevant to secretion-management strategies.

## Figures and Tables

**Table 1:** Peak inspiratory and expiratory flows and direction of secretion movement, with and without the in-line oscillatory device.

Mode/Setting	Without Device				With Device			
	Peak Insp (L/min)	Peak Exp (L/min)	Insp—Exp (L/min)	Movement (mm/min)	Peak Insp (L/min)	Peak Exp (L/min)	Insp—Exp (L/min)	Movement (mm/min)
VC-CMV PEEP 5	40	29	11	−4.33	40	47.0	−7	−6.00
VC-CMV PEEP 10	40	36	4	−11.67	40	49.4	−9.4	−9.00
VC-CMV PEEP 15	40	34	6	−5.67	40	49.8	−9.8	−7.00
APRV PHigh 20/PLow 0	40	43	−3	+4.33	41	45	−4	−3.33
APRV PHigh 25/PLow 0	42	45	−3	+6.33	50	55	−5	−6.33
APRV PHigh 30/PLow 0	52	62	−10	+14.33	55	66	−11	−0.33

Negative movement values indicate retrograde movement (toward the lung); positive movement values indicate antegrade movement (away from the lung). VC-CMV inspiratory flow was set at 40 L/min. Without the device, peak expiratory flow exceeded peak inspiratory flow only under APRV; with the device, peak expiratory flow exceeded peak inspiratory flow under both modes at every setting tested.

**Table 2:** Clinical studies examining APRV and VAP-related outcomes.

Study	Design (*n*)	PLow (cmH_2_O)	TLow Strategy	VAP Outcome	Key Confounders
Gonzalez 2010 [19]	International propensity-matched cohort (*n* = 234 vs. 234 (matched from 1228))	~7 (mean)	Not specified	APRV/BIPAP vs. unmatched cohort (6% vs. 10%, *p* = 0.03); No VAP difference in matched analysis (6% vs. 9%, *p* = 0.15)	APRV and BIPAP grouped as a single mode
Maxwell 2010 [20]	RCT, trauma respiratory failure (*n* = 63)	0	25–75% PEF; TLow 0.4–1.0 s	Numerically higher VAP in APRV (NS)	APRV arm sicker (APACHE II 20.5 vs. 16.9, *p* = 0.027); acknowledged randomization imbalance
Walkey 2011 [4]	Retrospective cohort, trauma with pulmonary contusion (*n* = 64)	1.2 ± 2.2	0.67 ± 0.18 s; strategy not specified.	HR 0.10 (0.02–0.58) favoring APRV	APRV had higher LIS (2.0 vs. 1.0, *p* = 0.0015) and more blood products (10 vs. 6 units, *p* = 0.04); transfusion is a recognized VAP risk factor.
Jain 2021 [21]	Retrospective COVID-19 cohort; conference abstract (*n* = 18 vs. 160)	Not reported	Not specified	No clear VAP signal (33.3% vs. 36.2%, *p* = 0.9)	APRV group small; tocilizumab more frequent in APRV, 33.3% vs. 10%, *p* = 0.015
Ergun 2023 [22]	Prospective observational, CARDS with prone positioning (*n* = 40)	~3	Not specified	Similar overall ICU-acquired infection (78% vs. 77%); VAP not separately reported	APRV combined with prone positioning
Naendrup 2024 [23]	Propensity-matched, CARDS (*n* = 40 vs. 40)	5 initially	50–75% PEF	No difference (62% vs. 50%, *p* = 0.26)	APRV used as rescue; severe ARDS twice as common in APRV arm; high outside-hospital transfer rate

ARDS = Acute Respiratory Distress Syndrome; BIPAP = biphasic positive airway pressure; CARDS = COVID-related ARDS; LIS = lung injury score; PEF = peak expiratory flow; RCT = Randomized Controlled Trial; VAP = ventilator-associated pneumonia.

## Data Availability

The raw data supporting the conclusions of this article will be made available by the authors on request.

## References

[1] Al-Khalisy H., Nieman G.F., Kollisch-Singule M., Andrews P., Camporota L., Shiber J., Manougian T., Satalin J., Blair S., Ghosh A. (2024). Time-Controlled Adaptive Ventilation (TCAV): A personalized strategy for lung protection. Respir. Res..

[2] Farkas J. PulmCrit—APRV: Resurrection of the Open-Lung Strategy?. https://emcrit.org/pulmcrit/aprv/.

[3] Papazian L., Klompas M., Luyt C.E. (2020). Ventilator-associated pneumonia in adults: A narrative review. Intensive Care Med..

[4] Walkey A.J., Nair S., Papadopoulos S., Agarwal S., Reardon C.C. (2011). Use of airway pressure release ventilation is associated with a reduced incidence of ventilator-associated pneumonia in patients with pulmonary contusion. J. Trauma.

[5] Nieman G.F., Gatto L.A., Andrews P., Satalin J., Camporota L., Daxon B., Blair S.J., Al-Khalisy H., Madden M., Kollisch-Singule M. (2020). Prevention and treatment of acute lung injury with time-controlled adaptive ventilation: Physiologically informed modification of airway pressure release ventilation. Ann. Intensive Care.

[6] Mahajan M., DiStefano D., Satalin J., Andrews P., Al-Khalisy H., Baker S., Gatto L.A., Nieman G.F., Habashi N.M. (2019). Time-controlled adaptive ventilation (TCAV) accelerates simulated mucus clearance via increased expiratory flow rate. Intensive Care Med. Exp..

[7] Camporota L., Rose L., Andrews P.L., Nieman G.F., Habashi N.M. (2024). Airway pressure release ventilation for lung protection in acute respiratory distress syndrome: An alternative way to recruit the lungs. Curr. Opin. Crit. Care.

[8] Daoud E.G., Farag H.L., Chatburn R.L. (2012). Airway pressure release ventilation: What do we know?. Respir. Care.

[9] Jain S.V., Kollisch-Singule M., Sadowitz B., Dombert L., Satalin J., Andrews P., Gatto L.A., Nieman G.F., Habashi N.M. (2016). The 30-year evolution of airway pressure release ventilation (APRV). Intensive Care Med. Exp..

[10] Zhou Y., Jin X., Lv Y., Wang P., Yang Y., Liang G., Wang B., Kang Y. (2017). Early application of airway pressure release ventilation may reduce the duration of mechanical ventilation in acute respiratory distress syndrome. Intensive Care Med..

[11] Lutz M.R., Charlamb J., Kenna J.R., Smith A., Glatt S.J., Araos J.D., Andrews P.L., Habashi N.M., Nieman G.F., Ghosh A.J. (2024). Inconsistent Methods Used to Set Airway Pressure Release Ventilation in Acute Respiratory Distress Syndrome: A Systematic Review and Meta-Regression Analysis. J. Clin. Med..

[12] Nieman G.F., Bates J.H.T., Andrews P.L., Rose L., Shiber J., Araos J., Aurelien L., Madden M., Manougian T., Satalin J. (2026). Expert recommendations for setting and adjusting airway pressure release ventilation based on clinical experience and basic science evidence. Front. Med..

[13] Spiegel R., Hockstein M. (2022). Airway Pressure Release Ventilation: A Field Guide for the Emergency Physician. Emerg. Med. Clin. N. Am..

[14] Daoud E.G., Yamasaki K.H., Nakamoto K., Wheatley D. (2018). Esophageal pressure balloon and transpulmonary pressure monitoring in airway pressure release ventilation: A different approach. Can. J. Respir. Ther..

[15] Lacherade J.C., De Jonghe B., Guezennec P., Debbat K., Hayon J., Monsel A., Fangio P., Appere de Vecchi C., Ramaut C., Outin H. (2010). Intermittent subglottic secretion drainage and ventilator-associated pneumonia: A multicenter trial. Am. J. Respir. Crit. Care Med..

[16] Osti C., Wosti D., Pandey B., Zhao Q. (2017). Ventilator-Associated Pneumonia and Role of Nurses in Its Prevention. JNMA J. Nepal Med. Assoc..

[17] Jam R., Mesquida J., Hernández Ó., Sandalinas I., Turégano C., Carrillo E., Pedragosa R., Valls J., Parera A., Ateca B. (2018). Nursing workload and compliance with non-pharmacological measures to prevent ventilator-associated pneumonia: A multicentre study. Nurs. Crit. Care.

[18] Pozuelo-Carrascosa D.P., Herráiz-Adillo Á., Alvarez-Bueno C., Añón J.M., Martínez-Vizcaíno V., Cavero-Redondo I. (2020). Subglottic secretion drainage for preventing ventilator-associated pneumonia: An overview of systematic reviews and an updated meta-analysis. Eur. Respir. Rev..

[19] Gonzalez M., Arroliga A.C., Frutos-Vivar F., Raymondos K., Esteban A., Putensen C., Apezteguia C., Hurtado J., Desmery P., Tomicic V. (2010). Airway pressure release ventilation versus assist-control ventilation: A comparative propensity score and international cohort study. Intensive Care Med..

[20] Maxwell R.A., Green J.M., Waldrop J., Dart B.W., Smith P.W., Brooks D., Lewis P.L., Barker D.E. (2010). A randomized prospective trial of airway pressure release ventilation and low tidal volume ventilation in adult trauma patients with acute respiratory failure. J. Trauma.

[21] Jain S., Londono C.G., Uribe-Marquez S., Nowak K. Conventional Protective LTV Versus APRV for Patients with Severe COVID-19 Disease. Proceedings of the Society of Critical Care Medicine 50th Critical Care Congress.

[22] Ergun B., Yakar M.N., Kucuk M., Baghiyeva N., Emecen A.N., Yaka E., Ergan B., Gokmen A.N. (2023). Combined Effects of Prone Positioning and Airway Pressure Release Ventilation on Oxygenation in Patients with COVID-19 ARDS. Turk. J. Anaesthesiol. Reanim..

[23] Naendrup J.H., Steinke J., Garcia Borrega J., Stoll S.E., Michelsen P.O., Assion Y., Shimabukuro-Vornhagen A., Eichenauer D.A., Kochanek M., Boll B. (2024). Airway Pressure Release Ventilation in COVID-19-Associated Acute Respiratory Distress Syndrome-A Multicenter Propensity Score-Matched Analysis. J. Intensive Care Med..

